# Computer-assisted technology for enhanced abdominal surgery

**DOI:** 10.1093/bjs/znab187

**Published:** 2021-05-26

**Authors:** L. O’Connell, D. C. Winter

**Affiliations:** Department of Surgery, St Vincent’s University Hospital, Dublin, Ireland

## Abstract

The application of computer-based technology to surgery has the potential to enhance the accuracy and outcomes of surgical procedures and perioperative care. Such innovative technologies include the integration of artificial intelligence into surgical decision-making, and the use of three-dimensional (3D) visual imaging, other real-time imaging techniques, and 3D printing technology.

**Summary box**

- Emerging computer-assisted technology has the potential to transform delivery of perioperative and intraoperative surgical care
- Examples include artificial intelligence, augmented reality, and molecular imaging and three-dimensional printing technologies
- Potential limitations include data protection and privacy concerns, the cost of developing and maintaining systems, and reliance on private industry

## Machine learning

Machine learning is an application of artificial intelligence that allows computer systems to learn from new information without requiring explicit programming to do so. Healthcare processes produce a massive volume of data, including individual-patient data, epidemiological data sets, and research literature. Clinicians evaluate such data to determine the best treatment strategy for each patient. However, it is beyond the capability of human cognitive function to fully acquire, constantly update, and instantly search such a massive data repository. In this respect, machine learning is uniquely suited to adaptation for use in healthcare. Such systems require massive data sets to gather sufficient information to enable them to provide accurate recommendations; they vastly surpass human capacity in terms of speed and volume of data that can be accessed.

There are two broad roles for machine learning in surgery: clinical decision support, whereby the system draws on its data set to assist in perioperative decision-making, and cognition-guided surgery, whereby a machine learning system provides assistance during a procedure.

Of all clinical tasks, performing surgery is a highly demanding, cognitively intensive process. The envisaged role of cognition-aided surgery is to develop a machine learning system that can ‘act in a similar way to a human assistant while permanently retaining knowledge which can be transferred, accumulated and used in future operations’^1^.

To date, machine learning systems have learned successfully to detect the steps of particular operations, and to guide the camera independently at laparoscopic surgery after being trained on intraoperative images at distinct steps of the procedure^2^.

## Computer-assisted surgical imaging

Augmented-reality imaging functions by creating a digital three-dimensional (3D) reconstruction of the patient’s anatomy based on preoperative imaging, and overlaying this model on to the corresponding anatomy during surgery^3^. Although used frequently in neurosurgery and orthopaedics, the tissue shift of deformable abdominal viscera presents difficulties with biomechanical modelling and real-time tracking^3^^,^^4^. Nevertheless, augmented reality facilitates virtual demonstration of critical structures, such as ureters and major vessels, during surgery. Virtual organ segmentation permits manipulation of the digital avatar, elements of the image can be subtracted or rendered transparent in order to visualize deeper structures, and virtual resection lines can be superimposed^3^. As fixed retroperitoneal structures are easier to reconstruct than mobile distensible viscera such as the small bowel and colon, augmented reality has been used as an aid in procedures such as adrenalectomy, pancreatectomy, and liver resections^3^^,^^4^.

In navigated surgery, the surgical instruments are combined with the preoperative imaging or intraoperative augmented-reality image and tracked during the procedure, thus providing a dynamic guidance system^1^^,^^4^. Navigation in abdominal surgery is subject to obstacles similar to those encountered in augmented reality, namely tissue shift and deformation. However, commercial platforms such as the CAS-One system™ (CAScination, Bern, Switzerland), which combines preoperative images with intraoperative ultrasonography, have been approved for clinical use and used successfully for complex liver resections. Navigation has also been applied successfully in urological procedures such as radical prostatectomy^3^^,^^4^.

Intraoperative molecular imaging is another computer-assisted imaging modality. Its primary potential is in surgical oncology. Occult tumours pose a challenge in terms of accurate localization and margin selection. Assessment of lymphatic metastasis is primarily performed before operation in abdominal surgery, in the absence of effective techniques for intraoperative evaluation of lymph node status. The aim of the molecular imaging techniques under development is to facilitate detection of small tumours and residual disease after neoadjuvant treatment, permit discrimination between neoplastic and normal tissue at a cellular level, and allow intraoperative identification of lymph node metastasis^5^.

Approaches include creation of fluorescence-tagged monoclonal antibodies to receptors known to be overexpressed in tumour *versus* healthy tissue. Several clinical trials are ongoing in this field, including a phase III trial in colorectal surgery using a fluorescence-tagged monoclonal antibody to the carcinoembryonic antigen receptor, and a phase I study of a tagged antiepidermal growth factor receptor antibody for detection of soft tissue sarcomas^6^.

Other methods include the development of optically active nanomaterial probes for intraoperative use. Nanomaterial-based probes can be designed to provide optical and metabolic features superior to molecular agents, and enhanced specificity for neoplastic tissue. Examples include: probes sized at 5–10 nm to optimize transport to, and limit extravasation from, regional lymphatics; fluorescent probes contained in a nanoparticle delivery system for improved depth penetration; and development of probes selectively taken up by neoplastic cells to facilitate precise margin selection^7^.

## Three-dimensional printing

3D printing is an additive manufacturing process in which a 3D virtual image of a target object is acquired by a computer, digitally reconstituted, and printed layer by layer^8–10^. Although to date it has been used predominantly to reproduce bony structures, 3D printing also has a number of useful applications in abdominal surgery^8–10^.

The primary application is in preoperative planning. It is most often used for reconstruction of the liver and kidney, as these are relatively fixed and constant in shape and location. Surgeons have report use of 3D-printed models as an aid in assessment of resectability and avoidance of hazards presented by aberrant anatomy^8–10^. Such models have been used for planning of renal and liver transplants, and visualization of abdominal vascular anatomy^8^^,^^9^. In the majority of the literature, surgeons reported that the 3D model offered value beyond radiological imaging alone, and in some instances altered the planned surgical strategy^8^^,^^9^.

**Fig. 1:**
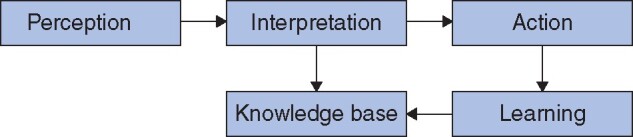
Principle of cognition-guided surgery The system uses perception from intelligent devices. It learns by feeding its experience back into the knowledge base. Reproduced from Kenngott *et al.*, *Innov Surg Sci* 2017;**2**:139–143, under Creative Commons Attribution License CC-BY 4.0.

Another application is in education. 3D models can enhance appreciation of complex anatomical information, particularly for students or trainees who are less familiar with radiological images. Printed models appear to be superior to both two-dimensional CT and virtual 3D reconstructions for this purpose^8^^,^^9^. They are also of relevance to patients as part of informed consent, and can provide a patient-specific visual aid to the surgeon in explaining the surgical strategy and potential complications.

In line with the shift towards personalized medicine, 3D printing may also be used to create implantable prostheses based on the patient’s own anatomy. Potential applications in abdominal surgery include creation of personalized grafts for endovascular aneurysm repair, where individualized implants have demonstrated superior outcomes to standard models^9^.

**Fig. 2:**
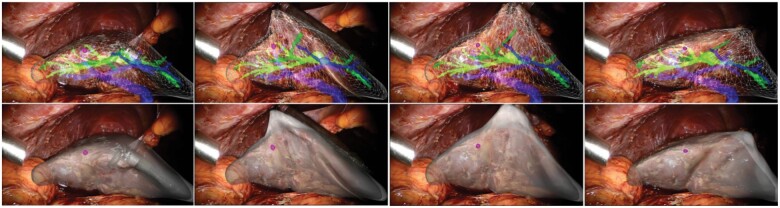
Selected frames of superimposition of digital model on to human liver during minimally invasive surgery The upper panels shown a heterogeneous model including parenchyma (white), biliary ducts (green), hepatic vein (blue), and a tumour (purple sphere). The lower panels show different visualization of superimposition. Reproduced with permission from Haouchine *et al.*, International Conference on Robotics and Automation, 2014.

**Fig. 3:**
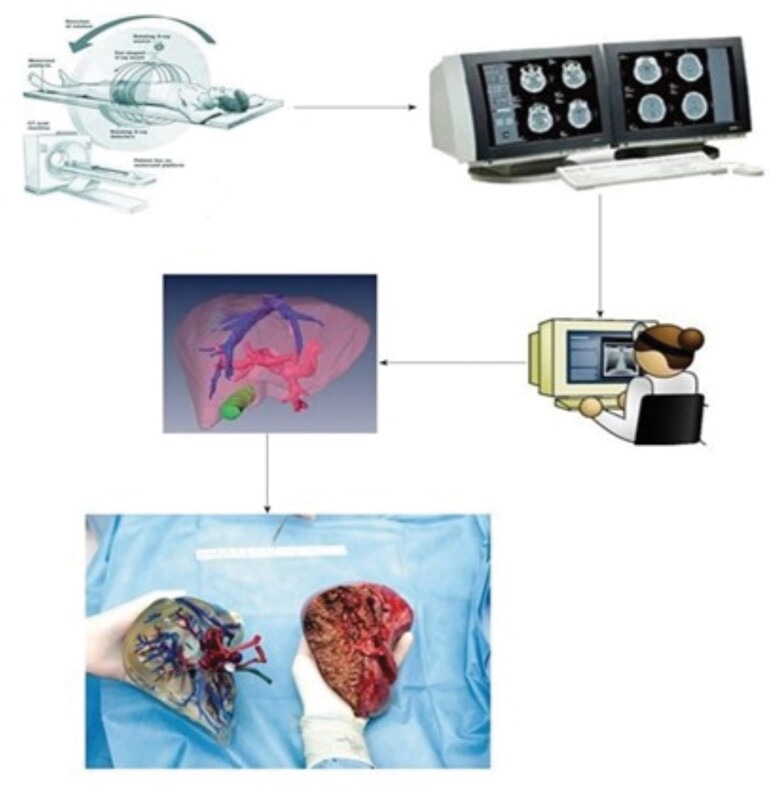
Process for creating three-dimensional printed liver model Reproduced from Bangeas *et al.*, *World J Hepatol* 2019;**11**:574–585, under Creative Commons Attribution License CC-BY 4.0.

##  

*Disclosure*. The authors declare no conflict of interest.
